# Promoting Health System Resilience Through Health Policy Reform for the Ageing Population of Japan: A Rapid Literature Review

**DOI:** 10.7759/cureus.90931

**Published:** 2025-08-25

**Authors:** Khang Duy Ricky Le

**Affiliations:** 1 Geelong Clinical School, Deakin University School of Medicine, Geelong, AUS; 2 Department of General Surgical Specialties, The Royal Melbourne Hospital, Melbourne, AUS

**Keywords:** ageing population, geriatrics patients, health financing, health policy, health system reform, health system resilience, japan health policy

## Abstract

Health system resilience encapsulates the ability of a health system to maintain appropriate standards of healthcare service delivery despite stressors to the system. Insults to the system can come through various forms, including natural disasters and, more recently, the influence of the coronavirus pandemic. Additionally, Japan faces a more insidious health system challenge through its rapidly developing ageing population, coupled with a lower funding base in light of this. Healthcare costs in Japan continue to rise disproportionately and are anticipated to do so due to care and costs related to ageing-related diseases. This rapid review explores the opportunity for health policy reform with respect to the financial and service delivery vulnerabilities of the Japanese health system to improve the capacity of the system to maintain health system resilience. This review does this through synthesising evidence on the current Japanese health finance system and payment models, and evaluates these in the context of current epidemiological and healthcare financing data. In doing so, the review identifies potential reform opportunities, particularly related to restructuring access to healthcare, promoting the use of generic pharmaceuticals, consolidating insurance, and adopting value-based payment models to improve health system resilience.

## Introduction and background

The idea of resilience has emerged with greater popularity in the last two decades. The concept features as a core component in United Nations (UN) policy, including the ‘Hyogo Framework for Action 2005-2015’ and the ‘Sendai Framework for Disaster Risk Reduction 2015-2030’, which call for an increased preparedness in disaster management [1,2]. Resilience has also been further endorsed within global health, with emphasis of resilience as a key component of health systems strengthening by the World Health Organisation (WHO), as well as in multiple UN Sustainable Development Goals; ‘resilience of the poor’ in target 1.5, ‘resilient agricultural practices’ in target 2.4 and ‘resilient to disasters’ in target 11b [3-5]. Notably, although the concept of resilience in these cases revolve more around disaster preparedness, global crises in the 21st century, including the 2011 Tohoku earthquake, the 2014-2016 Ebola Outbreak in West Africa, and the global COVID-19 pandemic, has led to a shift towards applying resilience to health systems across local, national and international jurisdictions [6].

Understanding health system resilience and, therefore, reform in this space is difficult due to the heterogeneous definitions of the concept. For example, Blanchett and James consider the idea of resilience as ‘the amount of change a (health) system can experience whilst maintaining the same controls on structure and function’ [7]. This idea is useful for public health practitioners in conceptualising the fundamental mechanics of resilience at an overarching level. Martineau adopts a health and person-centric definition in light of the Ebola crisis, considering resilience as a concept that involves not just the ‘system’, by acknowledging the contribution of individuals and their actions within the political and socioeconomic environment in times of stress [8]. In this way, resilience can be understood as the collective efforts of individual action in light of a common goal and within the specificity of challenges that threaten that specific system. Barasa et al. consider resilience based on the regular day-to-day functioning of a health system, emphasising the importance of access to essential resources required for everyday health service delivery and function [9]. At the heart of these definitions exists a common notion that health system resilience reflects the ability of the system to maintain functional homeostasis or core functions despite stress [10]. Furthermore, contemporary notions also emphasise health system resilience as the capacity of the system to undergo continual improvement to reduce vulnerability [6,11].

Building health system capacity for resilience requires intensive reform of system processes to adapt to the various acute and insidious challenges unique to the population. These ideas are well explored when focusing on the Japanese health system. Japan is often considered to have achieved and maintained universal health coverage. However, Japan continues to face challenges of an ageing population, declining birth rate, increasing social inequity, and higher rates of poverty [12]. Specifically, current statistics suggest Japan has the world’s largest aged population (29.1% of the population is aged 65 and over) with declining birth rates from one million births in 2016 to 750,000 in 2023 (a drop estimated to occur 12 years earlier than expected by the government) [13,14]. A consequence of this is the increased use of social and health services against the backdrop of decreasing taxpayers who fund these services [12]. Contemporary modelling suggests that there is expected to be a rise in the ageing rate to 36.3% by 2045, with an associated increase in healthcare costs to around Japanese Yen (JPY)89 trillion (more than 1.6 times the level of 2023) [15-17]. The Japanese health system, therefore, faces an insidious test to its ongoing resilience in light of growing expenditure and decreasing funding base, with an expected JPY27 trillion shortfall by 2040 unless reform occurs [15].

This rapid review aims to explore healthcare resilience in the setting of the ageing population of Japan through the health service financing, a key WHO building block of a health system [18]. In doing so, this review evaluates the impact of current challenges to the health system with the aim of proposing key avenues of health system reform as an instrument to promote resilience. 

## Review

Methods

This rapid review was conducted following a computer-assisted search of peer-reviewed and grey literature from MEDLINE (Medical Literature Analysis and Retrieval System Online) and Google Scholar databases as well as from key institutional documents authored by the Ministry of Health, Labour and Welfare of Japan, the Organisation for Economic Co-operation and Development (OECD), and the World Health Organization (WHO). Reference lists of select publications were also hand-searched to capture additional articles. Data were synthesised narratively.

Overview of health financing and expenditure in Japan

A key component of health system resilience is the ability of systems to maintain financial stability in everyday function, with or without external stressors [19]. Critical to policy reform in this space is to first gain an understanding of the key mechanics of the Japanese health system financing. Japan delivers a universal health insurance system, which has been praised for its ability to provide wide coverage with relatively low financial burden to residents [20]. The system adopts a Social Health Insurance (Bismarck) model, where residents are mandated to contribute 30% of medical costs [21]. This is offset by publicly sourced funds, combining public expenditure and insurance premiums to keep costs low for residents [21]. Health insurance in Japan is mandated and as per end of financial year 2021 data, consists either of employment-based insurance (~63% of population coverage) or residence insurance plans (Citizen Health Insurance for non/un-employed individuals covering 23% of the population and Health Insurance for Elderly plans covering 15% of the population ages > 75) [22]. There is mixed regional and municipal government regulation for insurance, with approximately 1400 employment-based plans and 47 residence-based plans specific to each prefecture [23,24].

To date, health costs for residents have been relatively contained. National data from the financial year 2022 suggests that 37.9% of costs were covered by public funds, 50.0% were covered by insurance premiums, and 11.6% were covered by individual out-of-pocket (OOP) payments [25]. OOP payments from 2019 accounted for 2.6% of household expenditure and were lower than the OECD average of 3.3% at this time [26]. Despite this, the increasing life expectancy of residents has led to a rapid emergence of a disproportionate ageing population requiring an increased demand on health services for the management of complex chronic diseases. In 2019, healthcare expenditure was 11.0% of Japanese Gross Domestic Product (GDP); the fifth highest expenditure across OECD nations and above the average of 8.8% [27]. Furthermore, the Japanese government highlights that spending increased by 28%, from JPY34.8 trillion to JPY44.4 trillion between 2008 and 2019, respectively, a value that exceeds the economic growth rate during this time of 8% (JPY508 trillion to JPY551 trillion) [28]. Japanese health spending continues to disproportionately rise, now accounting for 11.5% of GDP spending in 2023 (OECD average: 9.2%) [29]. Modelling data suggests that by 2065, 38.4% of the population is expected to be above age 75 (28.9% in 2021), with an estimated 25.5% at health risk (14.9% in 2021) [30]. Consequently, there is expected to be an associated increase in healthcare spending to account for these population changes.

This has multiple significant downstream effects, including pressure on funding for healthcare technologies, healthcare providers, medications, and, therefore, the capacity of health systems to function at baseline. Furthermore, at times of increased demand, such as with future disasters of any kind, there is the potential for these pressures to be exacerbated. This alludes to the discourse on resilience as defined by the aforementioned academics. In the absence of policy reform, the potential for these issues to arise is more likely [30]. Therefore, to promote health system resilience in light of these changes, policymakers are faced with the task of proactively addressing health spending through maintaining the well-being of the elderly and reforming health financing policy.

Influence of payment models on Japanese health system resilience

Payment for healthcare services in Japan is set by a national schedule and reimbursed on the basis of treatment. Similar to countries like Australia with the Diagnosis-Related Groups (DRG) system, inpatient hospital care is funded through a classification system of treatment episodes through the Diagnosis-Procedure Combination (DPC) system. Outside of this, funding predominantly occurs on a fee-for-service basis, with issues in this model including the possibility of incentivising perverse practices, including over-treatment, over-diagnosis, and polypharmacy; further increasing healthcare expenditure [30,31]. Japan has traditionally been able to maintain the cost of acute care to an acceptable standard through biennial revision of the fee schedule for inpatient hospital and specialist services, as well as lowering pharmaceutical prices [32]. Furthermore, Japanese health financing policy prohibits balance billing or charging gap fees for specialist and hospital services in order to prevent excessive healthcare costs to residents [23]. This has allowed the resilience of the health system through maintaining financial stability.

Uniquely, there is minimal distinction between primary care (such as through primary care, family medicine, or general practitioner services) and outpatient specialist care in Japan; both being delivered in clinics. In this way, the majority of primary care in Japan is privately owned and, like specialist outpatient clinics, is funded in a fee-for-service model. Despite the presence of public funding and health insurance, there is a reliance on payments from end consumers, including through OOP payments, co-payments, and co-insurance payments [23]. Residents are also expected to pay user charges for preventative health services such as cancer screening [23]. This leads to potential inequities in the provision of essential care in the community and therefore a test to the notion of resilience as maintaining everyday function.

To maintain universal care and provide resilience of the system by protecting the more vulnerable, the Japanese government introduces a safety net in its health funding policy. This safety net aims to protect those on the basis of income by limiting maximum monthly OOP based on income and age [23]. Similarly, there is an additional annual OOP ceiling also dependent on income and age to further protect individuals [23]. Furthermore, Japan’s public assistance program for low-income earners allows residents to avoid user charges [23]. Despite this, there is evidence to suggest that current supports are not sufficient to meet the demands of the population. An et al., in reviewing the nation’s public assistance program, identify that the program is ineffective due to a significant burden of disabled and sick individuals [33]. Moreover, inequity is seen in the elderly and low socioeconomic classes, who have been shown to forego basic dental care, a service covered on the national schedule, as a result of high co-payments [34].

Ageing-related chronic disease and multi-comorbidity have been estimated to account for 22% (USD3.5 trillion) of health expenditure [35]. This financial burden is expected to further increase with the ageing population. There have been two main areas of health policy reform in order to promote the resilience of the health system in light of these demands. The first is the introduction of the long-term care insurance (LTCI) system in the early 2000s. Residents aged 40 and over are invited to this insurance scheme, which seeks to fund anticipated nursing, aged care facility, or age-related disease care for those insured [36]. The scheme shares funding between the government (50%) and OOP insurance premiums with co-insurance rates of 10-30% (50%) [36]. In doing so, the government confers resilience to the system by improving future vulnerability through incentivising working-age citizens with more financial stability to fund the future age-related care. However, a deficiency of this program is its rapidly increasing funding requirements, with premiums paid by residents approximately doubling since its inception [37]. The second of these initiatives is the introduction of the National Mandatory Chronic Disease Prevention Program in 2008, following the new health reform law in 2006. The only of its kind worldwide, the program mandates residents in the age group of 40-74 to undergo annual health check-ups for lifestyle-related disease and metabolic syndrome, including diabetes, hypercholesterolaemia, hypertension, overweight/obesity, and associated metabolic syndromes [38]. In doing so, the system seeks to build resilience through primary and secondary prevention of chronic conditions, which are associated with a significant burden of disease and economic cost in society. At present, the economic impact of this program remains unclear. Key limitations of the program have been associated with challenges of poor health check-up rates, lack of integrative care infrastructure, and maldistribution of human resources to meet the demands of the population [38,39].

Opportunities for health finance reform to improve Japanese health system resilience

The Japanese health system is expected to face ongoing pressures due to a growing fiscal deficit in light of population ageing and increased social security payments. Given this, maintaining resilience demands a greater consideration of reform that considers improving efficiency in payment models and incentivising more considered health service utilisation without compromising health outcomes.

Micro-Environmental Level Opportunities

At a micro-environmental level, an opportunity exists in reshaping the way primary care is accessed. Currently, the function of tertiary hospitals, outpatient clinics, and general practitioners (GPs) is undifferentiated, which creates competition for service between these entities [40]. Unlike other jurisdictions, large hospitals are able to provide primary care. This is coupled with the higher degree of specialisation in GPs, who are able to offer both routine community-based primary care as well as varying degrees of secondary specialist care [40]. Furthermore, a large degree of these hospitals (including individual hospital beds) and clinics are owned privately by physicians and therefore the system is highly decentralised, with each individual entity deciding how to best invest capital. Reform that seeks to introduce a gatekeeper to specialist (secondary and tertiary) level care may potentially alleviate the burden of increasing health costs for the health system as well as its consumers [41,42]. These systems are common in high-income Western healthcare systems such as Australia, where referrals are required to access specialist secondary-level and above care. A key aspect of this approach would be the establishment of GP as an official subspecialty to allow patient-centred and accessible care in the community [43]. This feat is by no means simple and requires significant cross-collaboration between training colleges, healthcare providers, and government to best identify ways to accredit and recognise training of GPs without disincentivising uptake due to perceptions of wage penalty, prestige, or career prospects. Despite these theoretical outcomes, the literature suggests that referral-based systems alone do not directly influence healthcare spending, particularly in systems where primary care is funded by fee-for-service models [44,45]. These reforms, however, have potential for additional avenues of funding optimisation relevant to the Japanese health system, including improved management of healthcare resources, and have the potential to reduce supplier-induced demand [46]. This is particularly relevant with respect to the care of multi-comorbidity and elderly patients, who are expected to require complex care coordination and continuity of medical care.

Additionally, to better optimise funding at the micro-level, additional changes to health delivery practices must occur. In Japan, the usage rate of generic pharmaceuticals is low at an estimated 70% in 2020 compared to rates of 95% in the United States [47,48]. Overall, the cost of pharmaceuticals was JPY5.7 trillion in 2019 [49]. A key determinant of this is the lack of awareness of generic alternatives, including availability, accessibility, and therefore willingness to utilise these options [50,51]. Furthermore, the undifferentiation of prescribing and dispensing systems due to access models of care in Japan is also positively correlated with the use of brand-name alternatives [52]. Reform, therefore, should focus on promoting the use of generic drugs to alleviate the health cost burden on consumers and the system as a whole. In Japan, a pharmacist is able to dispense a generic drug unless a doctor has indicated a brand name [53]. Mechanisms to improve generic drug dispensing should seek to alter prescribing practices, such as through policies that promote the use of non-proprietary drug names rather than brand names. Reform in this space should also consider patient and provider education about generics, particularly related to safety and efficacy, to incentivise uptake [54]. Since 2020, policy inducements have led to Japan’s rates of generic drug use exceeding 90% in October 2024, indicating the benefits of policy reform in ameliorating financial challenges in healthcare [55].

Macro-Environmental Level Opportunities

At a macro-environmental level, reform that seeks to improve health financing are required to improve the resilience of the health system in light of population ageing challenges. There is a particular vulnerability in the insurance system with respect to health financing. Specifically, with the ageing population, the proportion of more senior enrollees has dramatically increased. This has led to pressures on health insurance fiscal balance with increasing expenditure and a lower funding base. This poses a significant issue in an environment with a vast number of insurers, like Japan. In particular, given that rates of premiums are determined by insurers independently, there is significant heterogeneity based on region/municipality. It is estimated that in some instances, these differences exceed 3% [56]. Others estimate that fragmentation in enrolment has led to situations where there is up to a three-fold difference in the proportion of income paid for premiums. Reform, therefore, is needed to equalise insurance contributions as a means to control costs to consumers [57]. In an attempt to alleviate such pressures, the government revised the national health insurance system in 2018, shifting certain financial responsibility from the city to the prefectural government [58]. Furthermore, they implemented subsidies within the system to alleviate costs for low-income families and the elderly. With over 3500 plans country-wide, there is a need for reform through consolidation of health insurance plans to equalise premiums at least at the prefectural level.

Furthermore, fee-for-service models disincentivise judicious healthcare spending, particularly in the decentralised and highly privatised system of Japan. In addition to considerations of primary care specialisation, there is a need to consider payment model reform models that best suit the ageing population and its complex care needs. To an extent, the LTCI was implemented for this purpose; however, it is challenged by increasing insurance costs. Shifting primary care funding from fee-for-service models to value-based models is a potential area to consider to improve the efficiency of funding without compromising health outcomes. Value-based programs (VBPs) are heterogeneous; however, key elements of VBP are an emphasis on outcome-based funding (i.e., paying for performance) and bundled-payment models for specific conditions [59,60]. By shifting fee-for-service models to VBP, there is the opportunity for policy makers to improve efficiency in funding through de-fragmentation and better integration of care coordination, and reducing supplier-demand servicing by promoting cost-conscious behaviour [61].

Aside from benefits in primary care coordination, application of CBP in secondary and tertiary services also has the potential to improve efficient healthcare resource allocation. This includes reducing redundancy in specialist input, more appropriate data sharing, and improved multidisciplinary collaboration. Attempts to adopt similar models have occurred in the context of enabling political interest and policy in this space. One particular area of reform is through the introduction of a community-based integrated care system, which aims to deliver a bundle of care for elderly patients, including acute care and longitudinal community-based chronic disease care, nursing care, preventive care, in addition to housing and living support [62,63]. Japan, however, remains challenged by the deeply ingrained structure of its health financing and service delivery models. In particular, the presence of a highly decentralised system, poorly defined primary care services, and service delivery largely driven independently within municipalities/regions/individual hospitals has led to maldistribution of appropriate resources within these pockets [64]. Downstream sequelae of this have been heterogeneity in community-based services and their quality in certain locations. In this way, systems-based reforms to health financing to promote resilience will have variable effects based on the capacity of each jurisdiction. Furthermore, there is a significant improvement that is required in terms of mobilising coordinated and integrated care, and similar controversy regarding adequate allocation of financial resources [64]. This is exacerbated by increasing public, insurance, and LTCI costs. Efforts at achieving resilience through a primary prevention approach, with efficient and coordinated care and value-based funding to meet the demands of the ageing population of Japan, are still in their infancy. Complexities occur with the design and implementation of appropriate policy and initiatives due to the independence of municipalities and regions in health delivery. There is a need for further research to identify areas of health finance policy optimisation and evaluation regarding how to best approach more integrated funding and care systems within the community.

## Conclusions

The Japanese health system faces a current and enduring challenge due to inherent vulnerability in health financing as a result of growing fiscal deficit in the context of a rapidly growing ageing population, increased social expenditure, and a lower funding base. Left unaddressed, this poses a significant threat to the resilience of the system in the future, including the capacity of the system to continue to provide universal health coverage to vulnerable populations, including low-income and elderly populations. There is a clear need for reform in payment models and access to care structures in the system to allow Japan to continue providing high-quality, safe, and affordable care in the face of these challenges.
